# Circulating exosome‐derived bona fide long non‐coding RNAs predicting the occurrence and metastasis of hepatocellular carcinoma

**DOI:** 10.1111/jcmm.14783

**Published:** 2019-12-07

**Authors:** Yunjie Lu, Yunfei Duan, Qinghua Xu, Li Zhang, Weibo Chen, Zhen Qu, Baoqiang Wu, Wensong Liu, Longqing Shi, Di Wu, Yan Yang, Donglin Sun, Xuemin Chen

**Affiliations:** ^1^ Department of Hepatobiliary Surgery The First People's Hospital of Changzhou The Third Hospital Affiliated to Soochow University Changzhou China; ^2^ Department of General Surgery Liyang People's Hospital Liyang China; ^3^ Department of Nephrology The First People's Hospital of Changzhou The Third Hospital Affiliated to Soochow University Changzhou China

**Keywords:** exosome, fingerprint, lncRNA, plasma, risk score analysis

## Abstract

Although the diagnosis and therapy approach developed, techniques for the early diagnosis of HCC remain insufficient which results in poor prognosis of patients. The traditional biomarker AFP, however, has been proved with low specificity. Circulating exosomal ncRNAs revealed different profiles reflecting the characteristics of tumour. In this study, we mainly focused on circulating exosomal ncRNAs which might be the fingerprint for HCC, especially for the diagnosis or metastasis prediction. A high throughput lncRNA microarray in exosomes extracted from cell‐free plasma was applied. The risk score analysis was employed to screen the potential exosome‐derived lncRNAs in two independent sets based on different clinical parameters in 200 paired HCC patients. After a multi‐stage validation, we finally revealed three lncRNAs, ENSG00000248932.1, ENST00000440688.1 and ENST00000457302.2, increased in HCC comparing with the both chronic hepatitis (CH) patients and cancer‐free controls. ROC curve revealed a higher sensitivity and specificity in predicting the occurrence of HCC from cancer‐free controls and CH patients with the area under curve (AUC) of 0.905 and 0.879 by combining AFP. The three lncRNA panel combined with AFP also indicted a fingerprint function in predicting the metastasis of HCC with the AUC of 0.870. In conclusion, ENSG00000248932.1, ENST00000440688.1 and ENST00000457302.2 might be the potential biomarker for the tumorigenesis prediction from CH patients or healthy controls and may also be applied for dynamic monitoring the metastasis of HCC.

## 1 INTRODUCTION

Hepatocellular carcinoma, also known as HCC, with the highly infection of HBV or HCV background in Chinese han population, is now listed as the most frequent liver cancer, with an increasing incidence over the last decades.^1^, ^2^ However, the diagnosis technologies for HCC in different stages has been improved. For example, imaging techniques such as US, CT and MRI, with a high resolution and was also recommended by guidelines.^3^, ^4^ However, for the nodules smaller than 2 cm in diameter in patients with liver cirrhosis, it was really difficult to distinguish.^5^ During the past decades, various biomarkers such as Alpha Fetal Protein (AFP), circulating miRNAs and lncRNAs have been proved as biomarker for HCC prediction. AFP was the traditional marker for the diagnosis or monitoring the recurrence for HCC; however, multiple studies have demonstrated the low specificity of AFP detection despite its high sensitivity.^6^, ^7^ A novel, minimally invasive indictor with high sensitivity and specificity is necessary for the HCC especially for the early diagnosis or dynamic monitoring.

Exosomes were defined as small membranous vesicles with a size of 30‐120 nm.^8^, ^9^ Circulating exosomes could be released into the extracellular environment through the fusion of multivesicular bodies approach with the membrane of certain body fluid such a serum or plasma.^10^, ^11^ It has been proved that ncRNA extracted from exosome could present a stable form to avoid the degradation induced by RNase, indicating they might be mediators for communications in different cells.^12^ Exosome‐derived non‐coding RNAs (ncRNAs) have been proved with different expression profiles which could indicate the characteristics of a certain tumour, or their function in tumour progression and metastasis.^13^ Various researches have proved that exosome‐derived ncRNAs could act as markers for the diagnosis and prognosis prediction in various kinds of human cancers.^14^, ^15^ However, most of the research mainly focused on the miRNAs. Although various proteins biomarkers such AFP, CEA, miRNA or lncRNAs have been isolated from exosomes as potential fingerprint for different approach, little is known about plasma exosome‐derived lncRNAs in HCC, little systematic study was conducted by using the high throughput detection of lncRNA, also many lncRNAs in exosomes have not been adequately investigated.

## 2 MATERIALS AND METHODS

### 2.1 Patients

Patients diagnosed with HCC and admitted to The First People's Hospital of Changzhou between October 2010 and September 2017 were enrolled in this study. Plasma samples were collected from these patients before surgical operation. Patients whom pathological diagnosis as either intrahepatic metastasis or portal vein tumour thrombus were regarded as metastasis HCC. This study was performed in accordance with the ethical guidelines of the Helsinki Declaration of 1975, as revised in 2008. All patients included in the study provided informed consent, and the study protocol was approved by the Institutional Review Board of The First People's Hospital of Changzhou. Experiments were undertaken with the understanding and written consent of each subject. Peripheral blood samples of patients were collected before operation. Blood samples were collected in a separate vacuum cube, followed by centrifugation at 1000 *g* for 10 minutes.

### 2.2 Isolation and identification of exosome‐derived RNAs from cell‐free plasma samples

The exosomes were extracted from plasma samples using the ExoQuick Exosome Precipitation Solution (System Biosciences). Briefly, 500 μL plasma was mixed with solution and then incubated for 30 minutes at 4°C. To validate the isolation of the exosomes from plasma, we performed the immunoblotting by using the exosome biomarkers such as CD9, CD63 and TSG101 for investigation. We also employed the calnexin as a negative control based on the little endogenous expression in exosome. The extracted exosomes were further detected on NanoSight LM10 instrument for the basic volume and concentration.

### 2.3 RNA extracting, microarray detection and data analysis workflow

The miRNeasy serum/plasma micro kit (Qiagen) was used for total RNAs isolation. The synthetic *Caenorhabditis elegans* miRNA (cel‐miR‐39; Applied Biosystems) was added to each sample as external reference.

A multiphase case‐control study was designed to identify the exosome‐derived lncRNA profile as a signature for HCC.

In the screening stage, RNA extracted from three HCC patients without metastasis (HCC‐N), three HCC patients with metastasis (HCC‐M), three patients diagnosed with chronic hepatitis (HCC) and three paired controls samples were also subjected for Human LncRNA Array v3.0 (Arraystar; Agilent) to screen the lncRNAs in different groups. Based on this, the RT‐qPCR was firstly used in the training set to further filter signals of the screened lncRNAs. The detailed steps for candidate screening and data filtering parameters were presented in Figure S1. The detailed primer sequence has been listed in Table S6.

### 2.4 Risk score analysis

The screening phase was divided into training set and validation set.

*Training set*

The training set contained 20 samples with random selection. The expression levels of these candidates were analysed in these samples, and the algorithms comparative $2^{-\Delta \Delta C_{t}}$ method were applied for analysing.

*Validation set*

The validation set enrolled 180 samples in each group. The case‐control study was designed to measure relative expression levels of the selected potential biomarkers candidates.

Risk score analysis was a traditional analysis to validate a certain biomarker. Here, data in the training set were performed to evaluate the associations between the concentrations of the plasma lncRNA expression levels. The upper 95% reference interval of each lncRNA value in controls was set as the threshold to code the expression level of the corresponding lncRNA for each sample as 0 and 1 in the training set. A risk score function (RSF) was defined according to a linear combination of the expression level for each lncRNA. For example, the RSF for sample *i* using information from four lncRNAs was as follows: rsfi = ∑3j‐1Wj.sij. In the above equation, sij is the risk score for lncRNA j on sample *i*, and Wj is the weight of the risk score of lncRNA *j*. We conducted the ROC analysis by using the total RSF value according to the case‐control group in the training set. We chose the value as the cut‐off value because the value of sensitivity + specificity was maximal.

### 2.5 Statistical analysis

If no special circumstances were declared, data were presented as mean ± SD. Chi‐square tests and Student's *t* test analysis of variance were used to evaluate statistical differences in demographic and clinical characteristics, respectively. Statistical analysis was performed using STATA 10.0 and presented with GraphPad Prism 5.0 software. Results were considered statistically significant at *P* < .05.

## 3 RESULTS

### 3.1 Clinical parameters analysis of patients enrolled in this study

A total of 600 patients including 200 HCC patients, 200 CH patients and 200 healthy controls were enrolled in this study. The detailed clinical information was listed in Table S1. All the HCC patients and CH patients was confirmed with HBV or HCV infection background. The tumours were smaller than 5 cm in 116 patients, 111 patients had multiple tumours, half patients were diagnosed with metastasis while only 14 patients with tumour capsular incomplete. The median AFP level was 32.7 ng/mL (Table S1). Besides, among the HCC patients, we divided into two groups according to the metastasis (HCC‐N and HCC‐M), each group enrolled 100 HCC patients. The clinical parameters were calculated and analysed including the tumour size, tumour number, tumour capsular, AFP value and TNM stage. As presented in Table S2, none of these was proved as significant.

### 3.2 Plasma exosomes identification and expression detection

**Figure 1:**
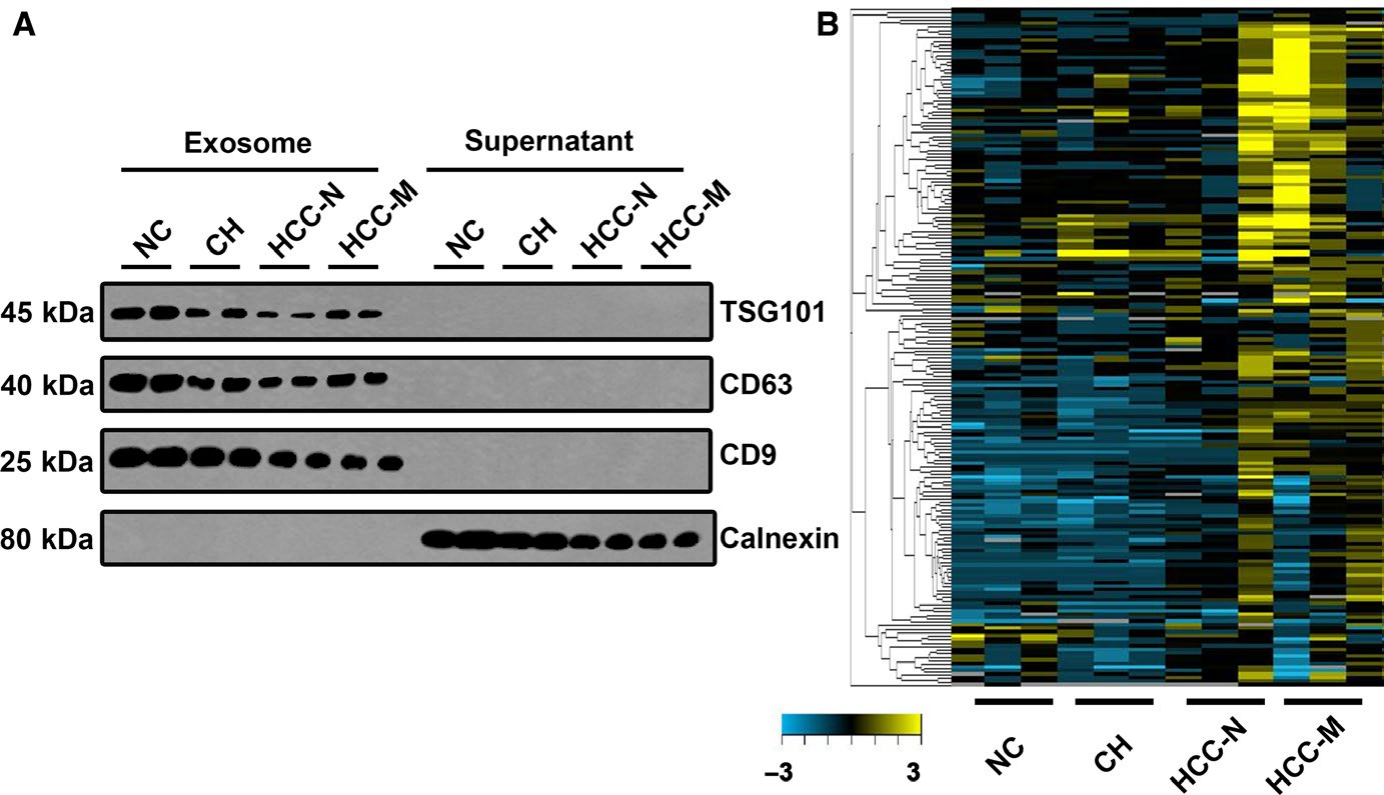
The expression landscape of circulating exosome‐derived lncRNAs in HCC patients. A, Expression of CD9, CD63, TSG101 and calnexin by Western blot. The expression of CD9, CD63, TSG101 and calnexin in isolated exosomal pellets from the sera of patients were compared with those of the supernatant. The expression of CD9, CD63, and TSG101 was present in the isolated exosomes, whereas calnexin, a negative marker of exosomes, was only expressed in the supernatant. B, Cluster analysis of the different expression of the lncRNAs in different groups. NC: normal control; CH: chronic hepatitis; HCC‐N: HCC without metastasis; HCC‐M: HCC with metastasis

Firstly, samples extracted from human plasma were detected by using exosome specific biomarker. As presented in Figure 1A, CD9, CD63 and TSG101 were used as positive controls for exosomes, and for calnexin, which is an integral protein of the endoplasmic reticulum and is not expressed in the exosome.^16^ We confirmed the high abundance of these factors in our exosome samples, while for the negative control, was only expressed in the supernatant.

**Figure 2:**
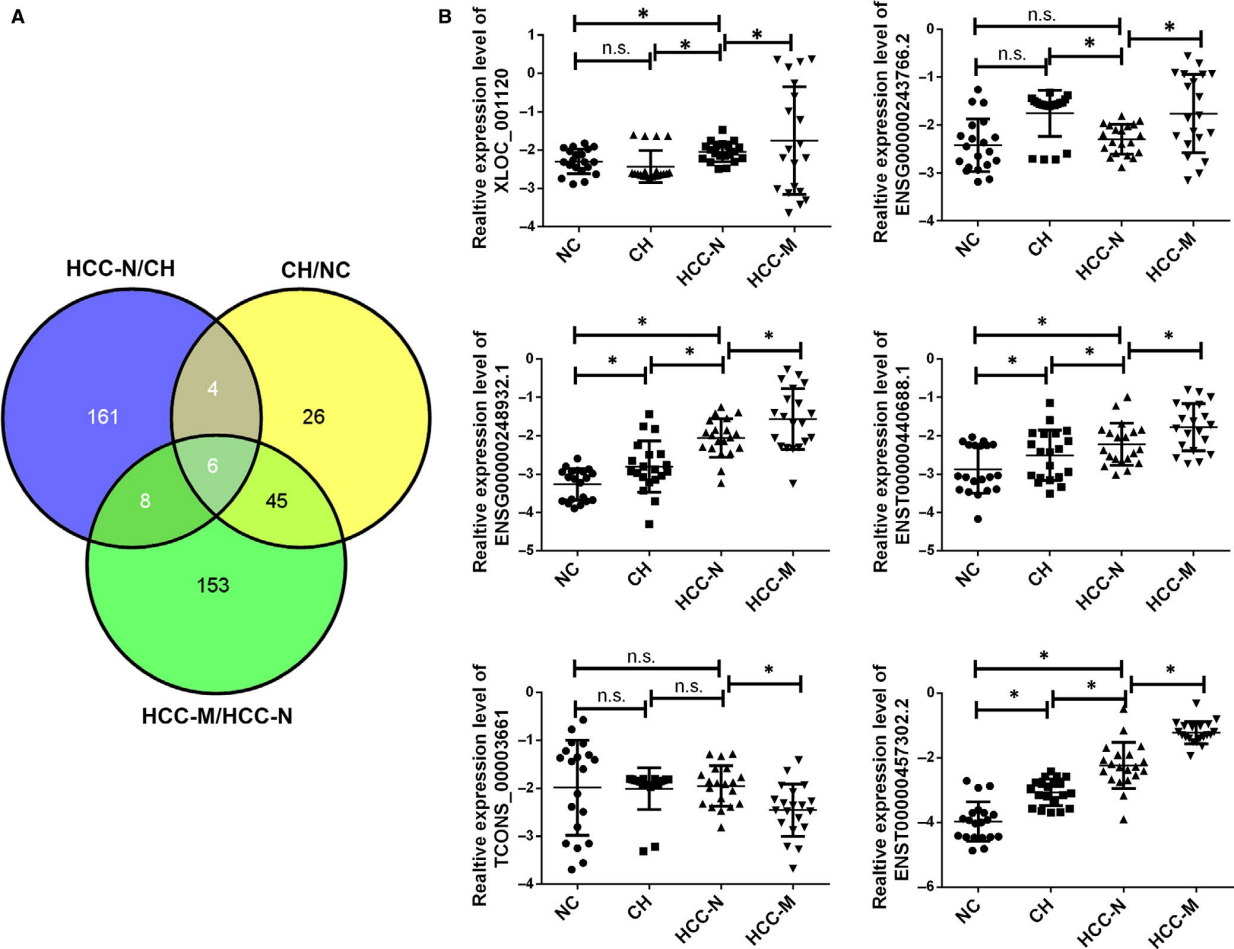
The screening work for candidate circulating exosome‐derived lncRNAs in the training set. A, Venny analysis of differently expressed lncRNA in NC, CH, HCC‐N and HCC‐M groups. B, The expression of lncRNAs was confirmed by RT‐PCR in groups. Data were presented as plot of the mean with SD with log‐transformed. * indicated *P* < .05, n.s. indicated no significance. CH, chronic hepatitis; HCC‐M, HCC with metastasis; HCC‐N, HCC without metastasis; NC, normal control

Based on this, we applied the total RNA extracted from the exosomes to the lncRNA microarray. Four groups including the healthy control (NC), CH group, HCC without metastasis (HCC‐N) and with metastasis (HCC‐M) were applied. Each group we enrolled three samples. Hierarchical clustering analysis and volcano plot distribution were used to sort the aberrantly expressed lncRNAs in different groups. As presented in Figure 1B, we obtained a different expression level of lncRNA in each group. We used the following parameters for further screening: (a) *P* value <.05; (b) CT value <35; (c) detection rate >75%. A total of 81 lncRNA transcripts were specifically increased in CH group comparing with NC group, 179 lncRNAs were collected in HCC‐N group by comparing with CH group while 212 lncRNAs were up‐regulated in HCC‐M group comparing with the HCC‐N group. In order to screen the biomarker predication the tumorigenesis and metastasis of HCC, the Venny analysis was applied and finally yielded 6 lncRNA candidates as listed in Figure 2A.

### 3.3 Identification of lncRNA genes from the training set and validation set

**Figure 3:**
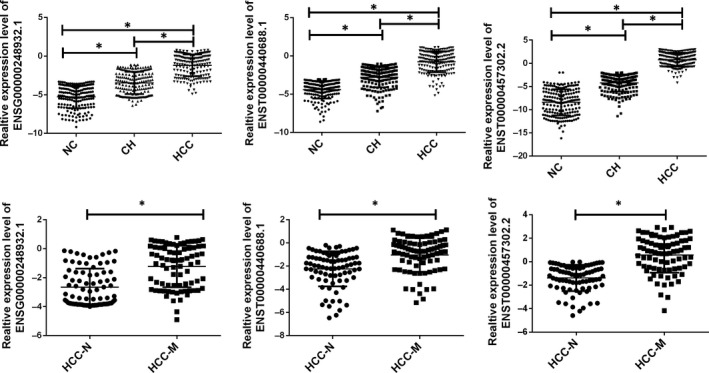
Relative expression of circulating exosome‐derived lncRNAs in validation set. Relative expression of circulating exosome‐derived lncRNAs in validation set. CH, chronic hepatitis; HCC‐M, HCC with metastasis; HCC‐N, HCC without metastasis; NC, normal control. Data were presented as plot of the mean with SD with log‐transformed. * indicated *P* < .05

To further analyse the aberrant expression of the six candidates, as summarized in the workflow, all analyses were performed in the training data set first and then validated in another set. The 20 samples in the training set were randomly selected from the 200 patients’ samples. We further examined these differentially expressed lncRNAs by RT‐qPCR in all the samples we enrolled. As presented in Figure 2B, the increased level of ENSG00000248932.1, ENST00000440688.1 and ENST00000457302.2 was confirmed in the larger sample size while the XLOC_001120, ENSG00000243766.2, AC058791.2 and TCONS_00003661 was revealed as no difference. Another 180 clinical samples were applied as the validation set. The three‐panel lncRNAs were also detected in these set, as presented in Figure 3, ENSG00000248932.1, ENST00000440688.1 and ENST00000457302.2 was confirmed with continuous increasing level in NC, CH and HCC groups. In addition, by comparing the HCC‐M with non‐metastasis group, we also obtained a up‐regulated level of all the three lncRNAs.

### 3.4 The association of three‐lncRNA signature and patient's occurrence/metastasis in the training set and validation set

With the risk score formula, we measured the three‐lncRNA expression signature risk score in the training set. The patients were then ranked according to their risk scores. By using the median risk score as cut‐off in the training set, the patients were divided into low‐risk and high‐risk groups. In the following analysis, we compared the function of the three lncRNA in predicting HCC from healthy controls, HCC from CH patients and HCC‐M from non‐metastasis patients. AFP was also used as an independent factor. The combination of the three lncRNAs was also calculated as an independent factor as well was the combination of the three lncRNAs and AFP.

Firstly, to explore the ability of three lncRNA in HCC patients from healthy controls, patients in the high‐risk group were labelled as HCC group while low‐risk was regarded as healthy controls. Based on this cut‐off, which was defined as the value of sensitivity plus specificity considered to be maximal, the positive predictive value (PPV) and negative predictive value (NPV) were 95% and 95% in the training set, respectively. As we used the same cut‐off value in the larger validation sets, the PPV and NPV were presented as 80%, 95%, respectively (Table S3).

**Figure 4:**
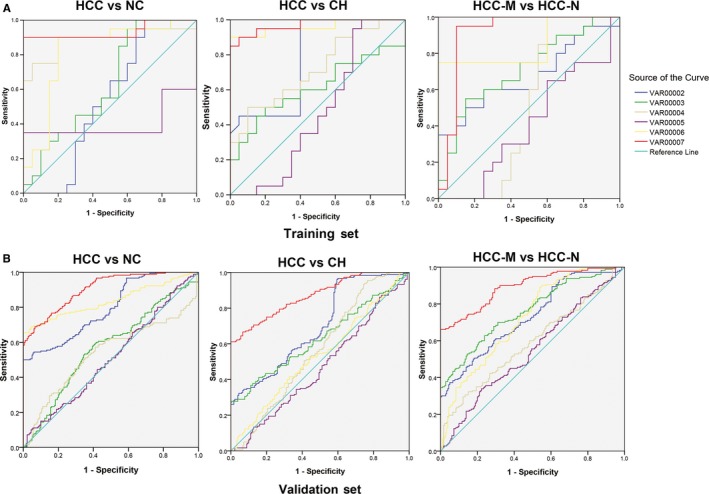
ROC analysis of the three‐potential fingerprint in different groups. A, The ROC curve for three‐potential fingerprint in the training set. B, The ROC curve for three‐potential fingerprint in validation set. VAR0002 to VAR0007 represent ENSG00000248932.1, ENST00000440688.1, ENST00000457302.2, AFP, the combination of the three lncRNAs, AFP plus the three lncRNAs. CH, chronic hepatitis; HCC‐M, HCC with metastasis; HCC‐N, HCC without metastasis; NC, normal control

We also used the ROC curves analysis to investigate the diagnostic sensitivity and specificity of the three‐lncRNAs signature for HCC. Each single lncRNA alone and the three merged factors were analysed, respectively. As presented in Figure 4A, the areas under the curve (AUCs) of ENSG00000248932.1, ENST00000440688.1, ENST00000457302.2, AFP, combination of lncRNAs and combination of lncRNAs and AFP were 0.530, 0.632. 0.883, 0.400, 0.818 and 0.833, respectively, in the training set. The values of AUC in validation set were 0.794, 0.571, 0.538, 0.510, 0.838 and 0.905, respectively, as presented in Figures S2 and S3.

Secondly, patients in the high‐risk group were labelled as HCC group while low‐risk was regarded as CH patients. Based on this cut‐off, which was defined as the value of sensitivity plus specificity considered to be maximal, PPV and NPV were 90% and 95% in the training set, respectively. Similarly, when the same cut‐off value was applied to calculate the risk score of samples in the larger validation sets, the PPV and NPV were 84% and 80%, respectively (Table S4).

We also used the ROC curves analysis to investigate the diagnostic sensitivity and specificity of the three‐lncRNAs signature for HCC. Each single lncRNA alone and the three merged factors were analysed, respectively. As presented in Figure 4B, the AUCs of ENSG00000248932.1, ENST00000440688.1, ENST00000457302.2, AFP, combination of lncRNAs and combination of lncRNAs and AFP were 0.776, 0.612. 0.720, 0.470, 0.960 and 0.970, respectively, in training set. The values of AUC in validation set were 00.699, 0.632, 0.565, 0.468, 0.534 and 0.870, respectively, as presented in Figures S2 and S3.

Thirdly, to explore the ability of three lncRNA in HCC‐M from non‐metastasis HCC patients, the patients in the high‐risk group were labelled as HCC‐M while low‐risk was regarded as HCC‐N. PPV and NPV were 90% and 95% in training set while 90% and 96% in validation set (Table S5).

The AUCs of ENSG00000248932.1, ENST00000440688.1, ENST00000457302.2, AFP, combination of lncRNAs and combination of lncRNAs and AFP were 0.672, 0.710. 0.505, 0.408, 0.850 and 0.910, respectively, in training set, and in the validation set, the AUC of which was 0.729, 0.762, 0.588, 0.546, 0.721 and 0.893, respectively, as presented in Figures S2 and S3.

### 3.5 Double‐blind test

Another 100 independent plasma samples were detected in a double‐blind fashion to validate the predictive ability of the three lncRNA. We used the same risk score formula to analyse the expression of the three exosome‐derived lncRNAs in those plasma samples and classifying them into a high‐risk group and a low‐risk group. Based on the pathologic diagnosis, the accuracy rate of the three exosome‐derived lncRNA profile as HCC signature from healthy controls, CH patients was 85.2%, 88.3%, while predicting the metastasis was 81.9%.

### 3.6 Stability expression of ENSG00000248932.1, ENST00000440688.1, ENST00000457302.2 in human plasma

**Figure 5:**
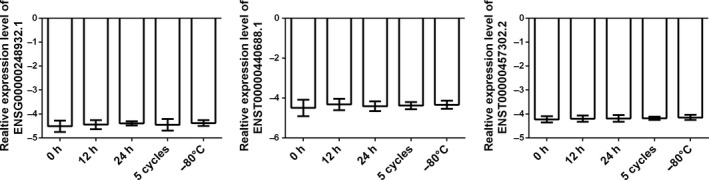
Expression stability examination of three circulating exosome‐derived lncRNAs. RT‐qPCR was applied for detecting the expression level of the three lncRNAs. Exosome‐derived RNA obtained from three healthy controls plasma samples were incubated at room temperature for 12 h, 24 h or subjecting it to up to three cycles of freezing and thawing. Data were presented as plot of the mean with SD. No significant difference was observed in each group

The expression of the three lncRNAs was detected in RNA sample extracted from four healthy controls and was incubated at room temperature for 12 hours, 24 hours, subjecting it to up to five cycles of freezing and thawing or under storage of −80°C for about 7 days. After that, the exosome was further extracted. All the process had minimal effects on the concentrations of the four lncRNAs, demonstrating that these lncRNAs were sufficiently stable in the exosome of human plasma (Figure 5).

## 4 DISCUSSION

The exploration of biomarker for HCC has been undertaking by multiple researchers over the past decades.^9^, ^17^, ^18^ The annotated biomarkers extracted from tumour tissues or cell‐free plasma has been proved might indicate the occurrence or recurrence of HCC.^19^, ^20^ HCC is one of the most common human malignant tumours worldwide and was proved with high mortality rates.^21^

Circulating ncRNAs have been well documented based on the crucial role during the pathogenesis and development of human cancer. Several ncRNAs have been developed as potential biomarkers for early screening.^22^ As we known exosome‐derived ncRNAs could remain a stable condition in plasma and exhibit different expression profiles representing the properties of cancer cells, researches hypothesized these exosome‐derived ncRNAs might serve as sensitive and non‐invasive biomarkers for both diagnostic and prognostic purposes. In recent years, increasing studies have focused on this field, resulting in a subclass of extracellular vesicles involved in intercellular communication released by most cell types and various body fluid.^23^ Cells can trigger cancer‐related disorders after the recognition and uptake of circulating exosome‐derived lncRNAs, providing indications for early tumour biopsy, diagnosis and treatment.^24^ Accumulated studies have implicated that exosomes play critical roles in the development and progression of malignant tumours. It has been confirmed tumour‐derived exosomes tumour antigens and promote tumour development.^25^ For example, Linc00974 has been identified as biomarker for HCC diagnosis which was involved in TGF‐beta‐associated pathway.^17^ Besides, long non‐coding RNA POU3F3 in plasma has also proved as a novel biomarker for diagnosis of oesophageal squamous cell carcinoma.^26^ Here in this study, exosome from plasma was first isolated and identified. Based on this, the exosome‐derived ncRNAs were extracted and were applied for the lncRNA microarray. The certain parameters were used for screening to guarantee the further validation. The risk score analysis was applied to test the diagnosis predicting ability for HCC from NC and CH patients as well as the metastasis of HCC. Finally, we obtained the ENSG00000248932.1, ENST00000440688.1, ENST00000457302.2 as candidate fingerprint. The ROC analysis with AFP as an independent biomarker also revealed that the three exosome‐derived lncRNAs panel indicated a higher sensitivity and specificity than AFP.

In conclusion, we identified three exosome‐derived lncRNAs, ENSG00000248932.1, ENST00000440688.1, ENST00000457302.2, as the potential fingerprints for the tumorigenesis prediction. Thus, we propose that this panel lncRNAs might be utilized to develop early diagnosis and invasive screening tools for HCC. More in‐depth studies are required to confirm the potential mechanism of this lncRNA in the development of HCC.

## CONFLICT OF INTEREST

The authors declared that they have no financial competing interest.

## AUTHOR CONTRIBUTIONS

YJL, DLS and YFD designed the study and drafted the manuscript; QHX, WBC, ZQ and BQW participated in data organization; YJL, LZ, WSL, LQS and DW collected the patients’ information; YY and XMC conducted the statistical analysis. All authors read and approved the final manuscript.
