# Supplementary material for: Circulating exosome‐derived bona fide long non‐coding RNAs predicting the occurrence and metastasis of hepatocellular carcinoma
Source: J Cell Mol Med. 2019 Dec 7;24(2):1311–8. doi: 10.1111/jcmm.14783 (PMC6991696; doi:10.1111/jcmm.14783)
Supplement: Supplementary file 1 [file JCMM-24-1311-s001.pdf]

Circulating exosome-derived bona fide long non-coding RNAs predicting the occurrence and metastasis of hepatocellular carcinoma

Yunjie Lu[1,*], Yunfei Duan[1, *], Qinghua Xu[2], Li Zhang[2], Weibo Chen[1], Zhen Qu[1], Baoqiang Wu[1], Wensong Liu[1], Longqing Shi[1], Di Wu[1], Yan Yang[3], Xuemin Chen[1, #], Donglin Sun[1, #]

1 Department of Hepatobiliary Surgery, The First People's Hospital of Changzhou, The Third Hospital Affiliated to Soochow University, Changzhou, Jiangsu, China

2 Department of General Surgery, Liyang People's Hospital, Liyang, Jiangsu, China.

3 Department of Nephrology, The First People's Hospital of Changzhou, The Third Hospital Affiliated to Soochow University, Changzhou, Jiangsu, China

Content:

Suppl Fig 1. Workflow for the exosome-derived lncRNA screening.

Suppl Fig 2. The detailed AUC value for each fingerprint in training set. VAR0002 to VAR0007 represent ENSG00000248932.1, ENST00000440688.1, ENST00000457302.2, AFP, the combination of the three lncRNAs, AFP plus the three lncRNAs. NC: normal control; CH: chronic hepatitis; HCC-N: HCC without metastasis; HCC-M: HCC with metastasis.

Suppl Fig 3. The detailed AUC value for each fingerprint in validation set. VAR0002 to VAR0007 represent ENSG00000248932.1, ENST00000440688.1, ENST00000457302.2, AFP, the combination of the three lncRNAs, AFP plus the three lncRNAs. NC: normal control; CH: chronic hepatitis; HCC-N: HCC without metastasis; HCC-M: HCC with metastasis.

Suppl Table 1. Clinicopathological features of surgical hepatocellular carcinoma (HCC), chronic hepatitis (CH) and cancer-free control samples.

Suppl Table 2. Clinicopathological features of surgical hepatocellular carcinoma (HCC) without metastasis(HCC-N) and with metastasis(HCC-M).

Suppl Table 3. Risk score analysis of in hepatocellular carcinoma (HCC) and cancer-free control plasma samples

Suppl Table 4. Risk score analysis of in hepatocellular carcinoma (HCC) and CH plasma samples.

Suppl Table 5, Risk score analysis of in hepatocellular carcinoma (HCC) metastasis and non-metastasis patients' plasma samples.

Suppl Table 6, Primers for Quantitative RT- PCR.

Supplementary Table1. Clinicopathological features of surgical hepatocellular carcinoma (HCC), chronic hepatitis (CH) and cancer-free control samples

	HCC	CH	Control	*P* value
**N**	200	200	200	
**Age Mean (SE) year**	55.43(12.4)	56.89(11.8)	56.79(11.6)	**0.44**[a]
**Sex (male/female)**	160/40	157/43	162/38	**0.82**[b]
**Differentiation grade**				
Well	96			
Moderate	61			
Poorly	43			
**Tumor Size(cm)**				
≤5cm	116			
>5cm	84			
**Tumor Number**				
Solitary	89			
Multiple	111			
**Tumor Capsular**				
Incomplete	14			
Complete	186			
**AFP(ng/mL)**	32.7(13.3)	28.3(11.2)	18.9(3.2)	**0.032**[a]
**TNM stage(I:II:III)**	98: 67:35			
**Metastasis**				
Yes	100			
No	100			
HBV/HCV				
Yes	200	200	/	**1.00**[b]
No	0	0	/	

[a] Student t-test.

[b] Chi-square test.

Supplementary Table2. Clinicopathological features of surgical hepatocellular carcinoma (HCC) without metastasis(HCC-N) and with metastasis(HCC-M).

	HCC- N	HCC-M	*P* value
**N**	100	100	
**Age Mean (SE) year**	56.12(11.5)	55.89(11.3)	**0.33**[a]
**Sex (male/female)**	78/22	82/18	**0.48**[b]
**Differentiation grade**			**0.69**[b]
Well	45	51	
Moderate	32	29	
Poorly	23	20	
**Tumor Size(cm)**			**0.39**[b]
≤5cm	55	61	
>5cm	45	39	
**Tumor Number**			**0.67**[b]
Solitary	43	46	
Multiple	57	54	
**Tumor Capsular**			**0.58**[b]
Incomplete	8	6	
Complete	92	94	
**AFP(ng/mL)**	31.8(10.8)	33.2(10.2)	**0.12**[a]
**TNM stage(I:II:III)**	45: 35:20	53:32:15	**0.47**[b]
**HBV/HCV**			**1.00**[b]
Yes	100	100	
No	0	0	

[a] Student t-test.

[b] Chi-square test.

Supplementary Table 3: Risk score analysis of in hepatocellular carcinoma HCC and cancer-free control plasma samples

Score	0-5.438	5.438-9.126	PPV [a]	NPV[b]
**Training set**			0.95	0.95
HCC	1	19		
Control	19	1		
**Validation set**			0.80	0.95
HCC	36	144		
Control	171	9		

[a] PPV, positive predictive value

[b] NPV, negative predictive value

Supplementary Table 4: Risk score analysis of in hepatocellular carcinoma HCC and CH plasma samples

Score	0-5.351	5.351-9.124	PPV [a]	NPV[b]
**Training set**			0.90	0.95
HCC	2	18		
CH	19	1		
**Validation set**			0.84	0.80
HCC	29	151		
CH	144	36		

[a] PPV, positive predictive value

[b] NPV, negative predictive value

Supplementary Table 5: Risk score analysis of in hepatocellular carcinoma(HCC) metastasis and non-metastasis patients' plasma samples

Score	0-1.128	1.128-3.339	PPV [a]	NPV[b]
**Training set**			0.90	0.95
Metastasis	2	18		
Non-metastasis	19	1		
**Validation set**			0.90	0.96
Metastasis	8	72		
Non-metastasis	77	3		

[a] PPV, positive predictive value

[b] NPV, negative predictive value

Supplementary Table 6. Primers for Quantitative RT- PCR

Gene name	All Patients	Sequence
XLOC_001120	Forward Primer	GCGGGCTTAGTAGCTTCAGG
	Reverse Primer	GTTGGGTAGTTGCCGTCTCC
ENSG00000243766.2	Forward Primer	TCCCTGTGCACCATTCATCC
	Reverse Primer	CAGGTCCGGTCCACAAAGAA
ENSG00000248932.1	Forward Primer	AACGAAGTGCCTAATCCCCG
	Reverse Primer	CTGGAGACTCGTTTCGCCTT
ENST00000440688.1	Forward Primer	AGCCACATGGCTCAGGATTC
	Reverse Primer	CGCCACTCCATAGTCACCAG
TCONS_00003661	Forward Primer	GGGTGACTCACTGAAGACGG
	Reverse Primer	ATAATCGCACAGGCAGAGGG
ENST00000457302.2	Forward Primer	TGTGACCTGAGGGACTGAAC
	Reverse Primer	AAGCCATTAGCCACAGGGAAA
ENST00000592884.1	Forward Primer	AATACAAGGATGAGGCCAGCTC
	Reverse Primer	TCTCCCTTAACGACGACTGC
ENST00000399447.3	Forward Primer	GCTTCTGGCTGCATTCTAGG
	Reverse Primer	CTCGCTTTCAGAAAACCACCC
ENST00000503186.1	Forward Primer	ACGTGCCTGTCTCCATCAAC
	Reverse Primer	GGGGTAGCAGAGACAAGGTG
GAPDH	Forward Primer	GGAGCGAGATCCCTCCAAAAT
	Reverse Primer	GGCTGTTGTCATACTTCTCATGG

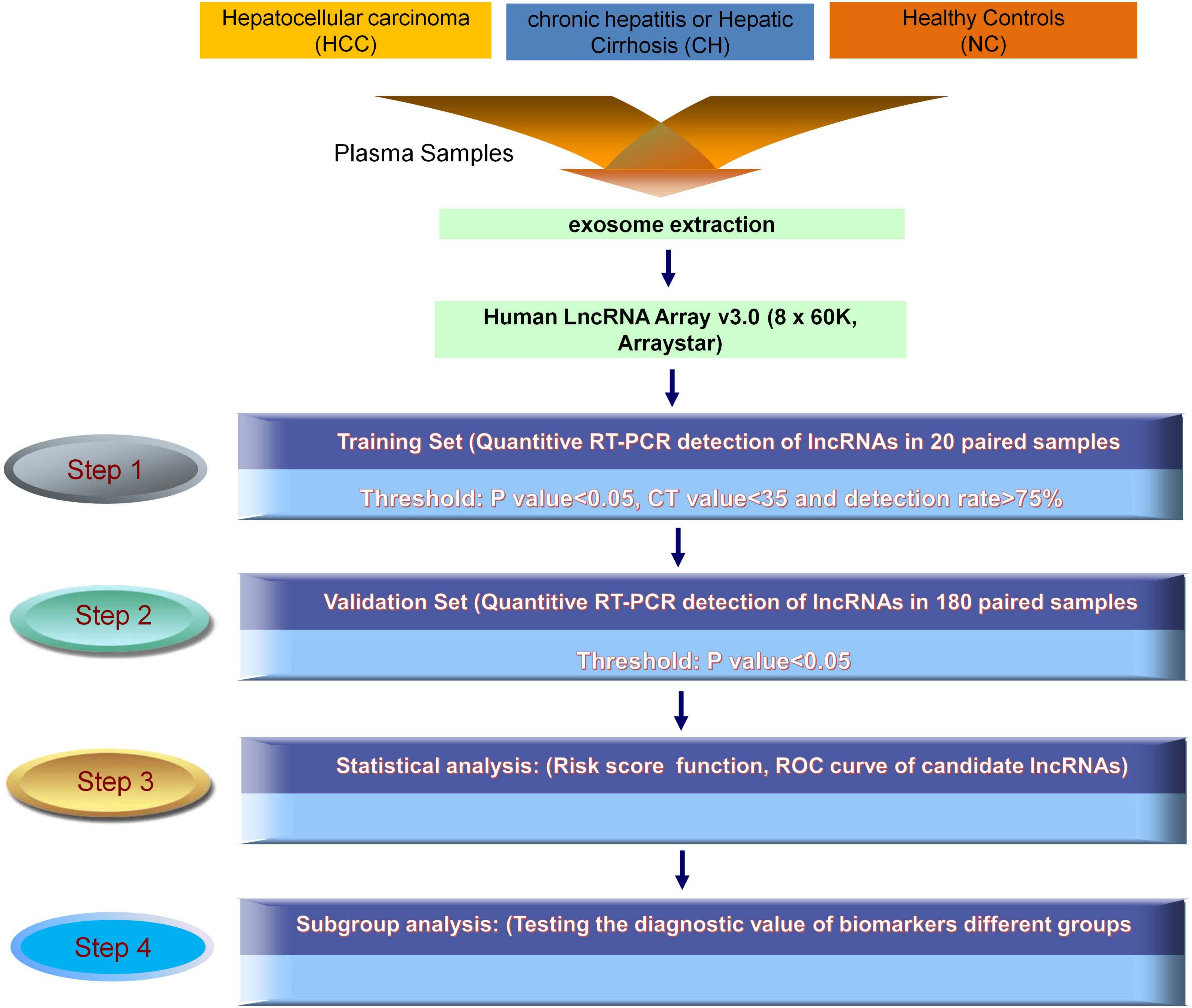
Hepatocellular carcinoma (HCC)
chronic hepatitis or Hepatic Cirrhosis (CH)
Healthy Controls (NC)
Plasma Samples
exosome extraction
Human LncRNA Array v3.0 (8 x 60K, Arraystar)
Step 1
Training Set (Quantitive RT-PCR detection of lncRNAs in 20 paired samples
Threshold: P value<0.05, CT value<35 and detection rate>75%
Step 2
Validation Set (Quantitive RT-PCR detection of lncRNAs in 180 paired samples
Threshold: P value<0.05
Step 3
Statistical analysis: (Risk score function, ROC curve of candidate lncRNAs)
Step 4
Subgroup analysis: (Testing the diagnostic value of biomarkers different groups

HCC vs NC

Area Under the Curve

Test Result Variable (s)	Area	Std. Error[a]	Asymptotic Sig.[b]	Asymptotic 95% Confidence Interval	
				Lower Bound	Upper Bound
VAR00002	.530	.097	.745	.340	.720
VAR00003	.632	.090	.152	.456	.809
VAR00004	.883	.060	.000	.765	1.000
VAR00005	.400	.103	.279	.198	.602
VAR00006	.818	.073	.001	.675	.960
VAR00007	.933	.047	.000	.840	1.025

a. Under the nonparametric assumption

b. Null hypothesis: true area = 0.5

HCC vs CH

Area Under the Curve

Test Result Variable (s)	Area	Std. Error[a]	Asymptotic Sig.[b]	Asymptotic 95% Confidence Interval	
				Lower Bound	Upper Bound
VAR00002	.776	.076	.003	.627	.926
VAR00003	.612	.092	.224	.431	.794
VAR00004	.720	.080	.017	.563	.877
VAR00005	.470	.097	.745	.280	.660
VAR00006	.960	.032	.000	.897	1.023
VAR00007	.970	.024	.000	.924	1.016

The test result variable(s): VAR00002 has at least one tie between the positive actual state group and the negative actual state group. Statistics may be biased.

a. Under the nonparametric assumption

b. Null hypothesis: true area = 0.5

HCC-M vs HCC-N

Area Under the Curve

Test Result Variable (s)	Area	Std. Error[a]	Asymptotic Sig.[b]	Asymptotic 95% Confidence Interval	
				Lower Bound	Upper Bound
VAR00002	.672	.087	.062	.502	.843
VAR00003	.710	.082	.023	.549	.871
VAR00004	.505	.104	.957	.302	.708
VAR00005	.408	.091	.317	.229	.586
VAR00006	.850	.065	.000	.722	.978
VAR00007	.910	.056	.000	.800	1.020

a. Under the nonparametric assumption

b. Null hypothesis: true area = 0.5

HCC vs NC (Validation Set)

Area Under the Curve

Test Result Variable (s)	Area	Std. Error[a]	Asymptotic Sig.[b]	Asymptotic 95% Confidence Interval	
				Lower Bound	Upper Bound
VAR00002	.794	.023	.000	.749	.839
VAR00003	.571	.030	.020	.512	.631
VAR00004	.538	.031	.215	.477	.599
VAR00005	.510	.030	.750	.450	.569
VAR00006	.838	.022	.000	.794	.881
VAR00007	.905	.015	.000	.876	.934

The test result variable(s): VAR00007 has at least one tie between the positive actual state group and the negative actual state group. Statistics may be biased.

a. Under the nonparametric assumption

b. Null hypothesis: true area = 0.5

HCC vs CH (Validation Set)

Area Under the Curve

Test Result Variable (s)	Area	Std. Error[a]	Asymptotic Sig.[b]	Asymptotic 95% Confidence Interval	
				Lower Bound	Upper Bound
VAR00002	.699	.027	.000	.646	.753
VAR00003	.632	.029	.000	.574	.689
VAR00004	.565	.030	.033	.505	.624
VAR00005	.468	.030	.291	.408	.527
VAR00006	.534	.030	.262	.475	.594
VAR00007	.870	.018	.000	.834	.905

a. Under the nonparametric assumption

b. Null hypothesis: true area = 0.5

HCC-M vs HCC-N (Validation Set)

Area Under the Curve

Test Result Variable (s)	Area	Std. Error[a]	Asymptotic Sig.[b]	Asymptotic 95% Confidence Interval	
				Lower Bound	Upper Bound
VAR00002	.729	.026	.000	.678	.780
VAR00003	.762	.025	.000	.713	.810
VAR00004	.588	.030	.004	.530	.647
VAR00005	.546	.030	.128	.487	.606
VAR00006	.721	.026	.000	.670	.773
VAR00007	.893	.016	.000	.861	.925

a. Under the nonparametric assumption

b. Null hypothesis: true area = 0.5
